# Emergence of Extensively Drug-Resistant *Neisseria gonorrhoeae,* France, 2023

**DOI:** 10.3201/eid3009.240557

**Published:** 2024-09

**Authors:** François Caméléna, Manel Mérimèche, Julie Brousseau, Mary Mainardis, Pascale Verger, Caroll Le Risbé, Elise Brottet, Alexandra Thabuis, Cécile Bébéar, Jean-Michel Molina, Florence Lot, Emilie Chazelle, Béatrice Berçot

**Affiliations:** Saint Louis–Lariboisière University Hospitals, Paris, France (F. Caméléna, M. Mérimèche, J. Brousseau, M. Mainardis, J.-M. Molina, B. Berçot);; French National Reference Center for bacterial STI, Paris (F. Caméléna, M. Mérimèche, J. Brousseau, M. Mainardis, B. Berçot);; Paris Cité University, Paris (F. Caméléna, M. Mérimèche, J. Brousseau, J.-M. Molina, B. Berçot);; Biogroup Oriade Noviale, Saint Martin d'Hères, France (P. Verger, C. Le Risbé);; National Public Health Agency, Lyon, France (E. Brottet, A. Thabuis);; National Reference Centre for Bacterial Sexually Transmitted Infections, Bordeaux, France (C. Bébéar);; University Bordeaux, Bordeaux (C. Bébéar);; Santé publique France, Saint-Maurice, France (F. Lot, E. Chazelle).

**Keywords:** gonorrhea, antimicrobial resistance, Drug-resistant, *Neisseria gonorrhoeae*, bacteria, penA-60.001, A2059G mutation, ST16406, sexually transmitted infections, surveillance, Ceftriaxone, Azithromycin, France

## Abstract

Since 2022, Europe has had 4 cases of extensively drug-resistant *Neisseria gonorrhoeae*, sequence type 16406, that is resistant to ceftriaxone and highly resistant to azithromycin. We report 2 new cases from France in 2023 involving strains genetically related to the 4 cases from Europe as well as isolates from Cambodia.

*Neisseria gonorrhoeae*, the causative agent of gonorrhea, remains a persistent global public health concern. *N. gonorrhoeae* is classified by the World Health Organization as a priority pathogen because of the emergence of multidrug-resistant isolates and rapid increases in antimicrobial drug resistance rates. Because of the frequency of resistance to cefixime, azithromycin, and fluoroquinolones, ceftriaxone is the recommended first-line treatment of *N. gonorrhoeae* (*1*). Four cases of extensively drug-resistant (XDR) *N. gonorrhoeae*, containing the *penA-60* allele and the A2059G mutation of 23S rRNA, belonging to sequence type 16406 have been reported in Austria, the United Kingdom, and France since 2022 (*2*–*4*). The enhanced gonococcal surveillance program in Cambodia identified 3 similar isolates during 2021–2022 (*5*). We report 2 new cases of XDR *N. gonorrhoeae* in France from 2023. Both patients gave consent for the publication of this report.

## The Cases

The patient in case 1 is a 40-year-old man living in the Rhône-Alpes region. He sought care in July 2023 for reported urethritis that occurred 15 days after unprotected sexual contact with a person living in Cambodia. The patient did not receive treatment in Cambodia before returning to France, and he had no sexual relations after his return. Laboratory testing of urethral samples was positive for gonococcal DNA and negative for *Chlamydia trachomatis*. *N. gonorrhoeae* was identified by using culture and designated as F95. The patient was treated with an intramuscular dose of ceftriaxone (1 g) during the visit. He attended a follow-up consultation with his general practitioner in September, during which control samples from his urethra, anus, and throat were collected. No *N. gonorrhoeae* DNA was detected from those samples; however, the patient still reported urethritis. Repeat laboratory testing was positive for *C. trachomatis*, and the patient was treated. 

The patient in case 2 is a 60-year-old man living with a partner in the Rhône-Alpes region. The patient sought care for reports of urethritis in August 2023. Symptom onset occurred 1 week after the patient had sexual contact with a sex worker in France. The patient had no documented travel to Asia. Laboratory testing of urine samples by using culture and PCR were positive for *N. gonorrhoeae*, designated F96, and *C. trachomatis*. In September, the patient was treated with ceftriaxone (1 g) for the *N. gonorrhoeae* infection and azithromycin (1 g) for the *C. trachomatis* infection. At a follow-up consultation a few weeks later, the patient reported no symptoms. Control samples from his urethra, anus, and throat were tested by using PCR and were negative for *N. gonorrhoeae* and *C. trachomatis*. 

The 2 *N. gonorrhoeae* isolates, F95 and F96, were sent to the national reference center for sexually transmitted infections in Paris for confirmation and genomic analysis. We conducted phenotypic antimicrobial drug susceptibility testing and found that both isolates were XDR (*6*). The isolates demonstrated resistance to penicillin G, cefixime, ceftriaxone, ciprofloxacin, azithromycin, tetracycline, and doxycycline; both isolates were susceptible to ertapenem and aminoglycosides (Table). We conducted whole-genome sequencing by using an Illumina MiSeq (Illumina, https://www.illumina.com) system (300 cycles, 2 × 150 bp). We performed bioinformatic analysis as previously described (*4*). The sequences obtained are available from the European Nucleotide Archive and GenBank (accession no. PRJNA1082518). 

**Table:** MICs and molecular resistance mechanisms of extremely drug-resistant *Neisseria gonorrhoeae* strains F95 and F96, recovered from 2 patients in France, 2023*

Antimicrobial	F95 MIC, mg/L	F96 MIC, mg/L	EUCAST interpretation	Molecular mechanism(s) of resistance
Penicillin	>32	>32	R	*bla*_TEM-135_
Cefixime	1	1	R	*penA* 60.001 (mosaic; A311V; T483S); *mtrR*, A39T; *ponA*, L421P; *porB* structure, porB1a
Ceftriaxone	0.25	0.25	R	
Cefotaxime	0.5	0.5	R	
Ertapenem	0.032	0.016	NA	NA
Azithromycin	>256	>256	R (high-level)	23S rRNA, A2059G; *mtrR*, A39T
Tetracycline	32	32	R (high-level)	*tetM*; *rpsJ*, V57M; *mtrR*, A39T
Doxycycline	16	16	R	
Ciprofloxacin	2	4	R	*gyrA*, S91F and D95A; *parC*, S87R
Spectinomycin	16	16	S	NA
Gentamicin	8	8	NA	NA
Rifampin	0.125	0.064	NA	NA
*MIC testing done using ETEST (bioMérieux, https://www.biomerieux.com). EUCAST, European Committee on Antimicrobial Susceptibility testing; NA, not applicable because of a lack of breakpoints for interpretation; R, resistant; S, susceptible.				

We aligned the genomes of F95 and F96 with ceftriaxone-resistant strains previously isolated in France (WHOY [F89], F90, F91, and F92), Japan (WHOX), and the United States (LRRBGS-1327). We also aligned the F95 and F96 genomes with strains highly resistant to azithromycin and resistant to ceftriaxone recently found in the United Kingdom (WHOQ and H22-494), Austria (AT158), France (F93 and F94), Cambodia (22R655558T, 22R655567T, and 22R655494S), and Australia (A2735 and A2543) (*2*–*5*,*7*–*12*). We used ParSNP v.1.2 (https://github.com/marbl/parsnp) to correct the alignments for recombination. We then constructed a maximum-likelihood phylogeny for core-genome comparisons. We visualized the comparisons by using iTol (https://itol.embl.de), as previously described (*4*, *7*). The whole-genome sequencing analysis revealed that isolates F95 and F96 belonged to sequence type 16406. The F95 and F96 strains were assigned to sequence type 22862 by using *N. gonorrhoeae* multiantigen sequence typing (*porB* 822, *tbpB* 294) and 5793 by using *N. gonorrhoeae* sequence typing for antimicrobial resistance (profile no. 60.001_89_13_1_7_3_1).

Our molecular analysis of isolates F95 and F96 revealed several mutations associated with antimicrobial resistance (Table). Both isolates contained the mosaic allele *penA-60.001*, which encodes a penicillin binding protein 2 with amino-acid substitutions (A311V, T4835) that impair the binding of extended-spectrum cephalosporins (ESCs). This impairment renders the isolates resistant to ESCs. F95 and F96 contain the A39T mutation in the *MtrR* repressor gene, without promoter deletions, which causes an overexpression of the MtrCDE efflux pump and an increase in the MIC of ESCs. The isolates produced TEM-135 penicillinase with a M182T substitution known to increase the stability of the enzyme, which typically proceeds additional mutations that extend the range of resistance to ESCs. In addition, F95 and F96 carried the A2059G mutation in the 4 alleles of the 23S rRNA-encoding gene, conferring high levels of resistance to azithromycin (*6*). The quinolone resistance-determining regions of F95 and F96 contained S91F and D95A substitutions in *GyrA* (a subcomponent of DNA gyrase), and an S87R substitution in *ParC* (a component of topo-isomerase IV). Those substitutions are responsible for high level resistance to ciprofloxacin. Finally, both strains contained the *tet(M)* gene and a V57M amino-acid substitution in the *rpsJ* gene (encoding the S10 ribosomal protein), conferring a high level of tetracycline resistance.

The F95 isolate had no single-nucleotide polymorphism relative to the F96 isolate. Both isolates clustered with other XDR *N. gonorrhoeae* isolates that contained the *penA-60.001* allele and the A2059G mutation in 23S rRNA, indicating a common evolutionary origin (Figure). F95 and F96 were closely related to the recently reported azithromycin-resistant and ceftriaxone-resistant 22R655567T XDR isolates from Cambodia, only differing by 113 single-nucleotide polymorphisms. On the phylogenetic tree, the F95 and F96 isolates belonged to the same lineage as the XDR isolates from Cambodia. This lineage is further divided into multiple sublineages: the A2543 XDR clone, which has been reported to be spreading internationally; the sublineage including AT159, H22-494, and F93; the sublineage F94; the sublineage 22R655558T; and the sublineage including F95, F96, 22R655567T, and 22R655494S (Figure) (*2**–**5**,**10*).

**Figure:**
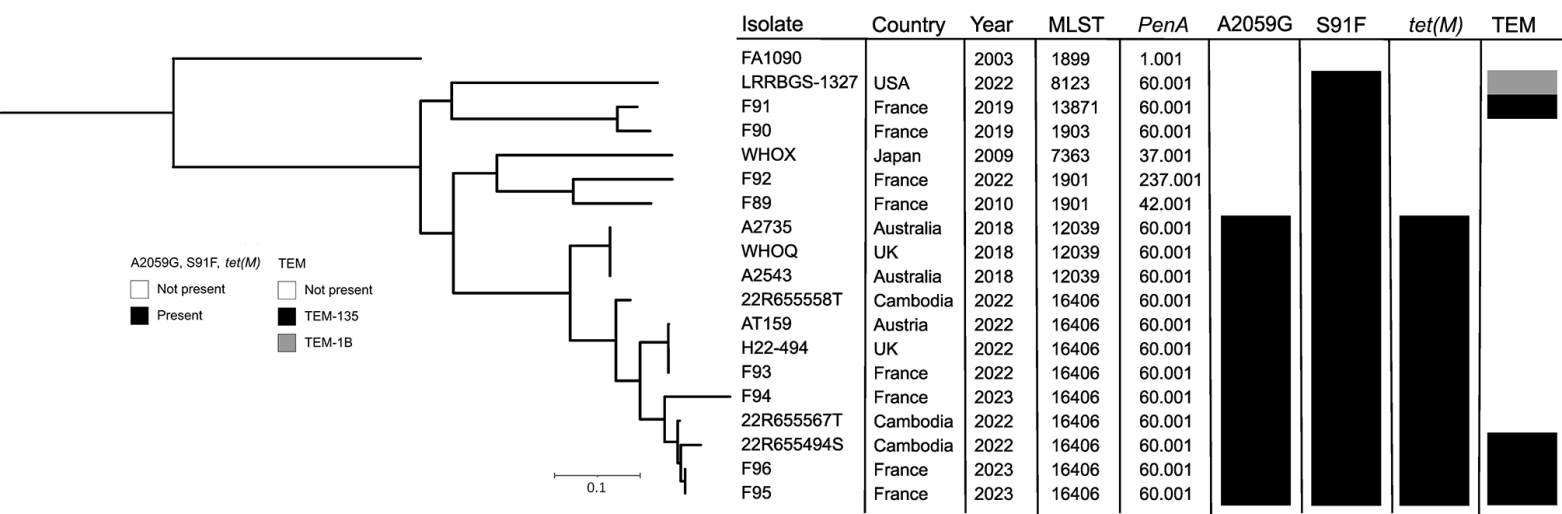
Phylogenetic tree of 19 *Neisseria gonorrhoeae* isolates, including 2 from patients in France, compared on the basis of their main resistance determinants. The phylogenetic tree was built from 10,907 total core single-nucleotide polymorphism positions. The F95 and F96 isolates from 2 patients in France were compared with ceftriaxone-resistant and extensively drug-resistant *N. gonorrhoeae* isolates from Europe, Australia, Cambodia, Japan, and the United States. The tree is rooted on *N. gonorrheae* FA1090, a laboratory reference strain. Scale bar indicates the branch length corresponding to genetic change.

The patients in both cases were successfully treated with 1 g ceftriaxone, which suggests that EUCAST breakpoints (https://www.eucast.org) for ceftriaxone resistance (MIC >0.125 mg/L) might be too low for genital infections (*13*). In case 2, the patient likely contracted XDR *N. gonorrhoeae* infection through sexual contact with a sex worker. However, no additional information was obtained about the sex worker, and our investigation did not establish a link to case 1. The XDR *N. gonorrhoeae* isolates also contained the *tetM* gene, which confers resistance to tetracycline and doxycycline. Several public health departments in California, USA, recommend the use of doxycycline postexposure prophylaxis in men who have sex with men, and the indirect selection of *N. gonorrhoeae* containing *tet(M)* because of the use of doxycycline postexposure prophylaxis is possible (*14*,*15*). 

## Conclusions

We report 2 cases of XDR *N. gonorrhoeae* strains from France. These strains are highly resistant to azithromycin, resistant to ceftriaxone, and genetically related to isolates from Cambodia. Our findings raise concerns about the spread of XDR *N. gonorrhoeae* in Southeast Asia. The emergence and spread of XDR *N. gonorrhoeae* isolates suggest the need to reconsider current recommendations for the first-line treatment of gonococcal infections. Novel and effective therapies against these XDR isolates are required, as is the need for international collaboration to monitor antimicrobial resistance.
